# Expanding the Capabilities of Portable Mapping in Macroscopic External Reflection FT-IR through a Targeted Data-Driven Spectral Enhancement and Denoising Strategy

**DOI:** 10.1021/acsmeasuresciau.5c00163

**Published:** 2026-02-12

**Authors:** Zelan Li, Emilio Catelli, Jošt Stergar, Matija Milanič, Roberto Sáez-Hernández, Silvia Prati, Paolo Oliveri, Giorgia Sciutto

**Affiliations:** † Department of Chemistry “Giacomo Ciamician”, 9296 University of Bologna, Via Guaccimanni, 42, Ravenna 48121, Italy; ‡ Faculty of Mathematics and Physics, 37663 University of Ljubljana, Ljubljana SI-1000, Slovenia; § Jožef Stefan Institute, Ljubljana SI-1000, Slovenia; ∥ Department of Analytical Chemistry, Faculty of Pharmacy, 16781 University of Valencia, Dr. Moliner, 50, Burjassot, Valencia 46100, Spain; ⊥ Department of Pharmacy (DIFAR), University of Genova, Genova I-16148, Italy

**Keywords:** denoising, data-driven enhancement, spectral imaging, portable mapping system, external reflection Fourier transform infrared

## Abstract

Reliable spectral quality is essential for extracting meaningful information from infrared reflectance data, particularly when using portable systems with limited scan numbers. This study presents a data-driven spectral enhancement workflow designed to improve the interpretability of portable macroscopic external reflection Fourier Transform Infrared (MA-rFT-IR) mapping systems developed by the Authors, operating in the near- and mid-infrared (NIR–MIR) ranges. Despite the growing use of reflectance imaging spectroscopy, limited attention has been devoted to the development of robust denoising strategies capable of minimizing noise and unwanted variability while preserving spectral quality and enabling more reliable and accurate data analysis. This study proposes a broadly applicable processing framework aimed at enhancing the efficiency and performance of reflectance-based spectral analysis. Denoising methods including Savitzky–Golay filtering and wavelet- and PCA-based denoising were tested and evaluated individually and in combination. Quantitative performance was assessed using arccosine similarity (ACOS) and derivative-based root-mean-square error (dRMSE) metrics across selected spectral regions of interest, with a derivative ACOS (dACOS) index applied to monitor band-shape variations. The evaluation results were integrated through Pareto analysis to identify the optimal trade-off between noise reduction and spectral-feature preservation. Application of the proposed approach to a multilayered painting mock-up demonstrated that the enhancing spectral data workflow preserves key diagnostic features revealing subtle spectral bands. Furthermore, applying multivariate curve resolution-alternating least-squares (MCR-ALS) to the denoised data enabled chemically meaningful separation of complex overlapping signals, improving the interpretability of compositional information compared with traditional denoising methods and data processing. The workflow strengthens the analytical reliability of low-scan reflection-mode data and provides a transferable framework for optimizing denoising strategies in portable infrared applications.

## Graphical abstract

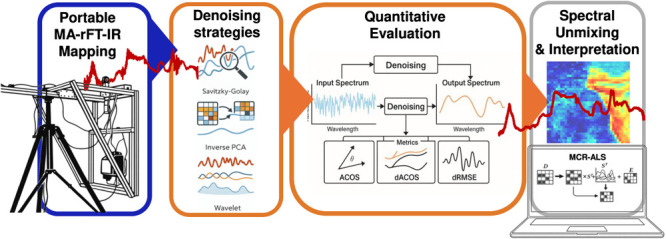

## 1 Introduction

Reliability of spectral data is a key factor in the chemical characterization of materials. Nevertheless, spectral quality can be significantly affected when data are collected under noncontrolled environments, such as *in situ* analyses or with portable instrumentation.^1,2^ In such cases, the combination of low signal averaging and complex reflection geometries can introduce substantial spectral noise and baseline fluctuations, hindering the interpretation and quantitative comparison of the acquired data.^3,4^ Addressing these limitations requires analytical strategies that enhance the spectral quality without altering the original signal or compromising chemical meaning. This study presents a data-driven workflow that integrates optimized denoising and quantitative evaluation methods to improve the reliability of reflection-mode infrared mapping. Although the approach was developed for the analysis of painted surfaces, it is broadly applicable to other systems where rapid or noninvasive spectral acquisition is required.

Fourier Transform Infrared (FT-IR) spectroscopy in reflection mode (rFT-IR) has long been recognized for its effectiveness in material analysis due to its ability to probe molecular bond vibrations on the sample surface. In particular, external reflection (ER) FT-IR enables noncontact examination of highly absorbing or opaque samples, overcoming challenges associated with conventional transmission modes.^5,6^

In heritage science, the constraints of noninvasiveness and *in situ* applicability are particularly well fulfilled by portable ER-FT-IR.^4–6^ More recently, the growing demand to analyze large heterogeneous objects, such as mural and easel paintings, has further increased the necessity for macroscopic analytical approaches capable of characterizing chemical compositions across extensive areas.^7^

A practical response is mid-infrared (MIR) reflectance imaging spectroscopy, which records infrared spectra at every pixel and, when equipped with high-throughput detectors, can collect large infrared images rapidly with high spectral quality. However, the high cost of the instrumentation, particularly for MIR focal plane arrays (FPAs),^8^ limits the widespread use. Beyond cost, most MIR imaging instruments cover only a portion of the MIR window: the long-wave instrument of Rosi et al. operates in the 826–1300 cm^–1^ fingerprint range, leaving higher-frequency bands inaccessible, while the Daveri et al. spans 2700–5500 nm (≈ 3700–1800 cm^–1^), missing the lower-wavenumber fingerprints.^9,10^ Furthermore, certain imaging approaches rely on illumination sources, which pose the potential risk of heating the delicate surface of cultural heritage objects.

Implementing point-by-point mapping can offer an affordable route for generating chemical distribution maps, but it often requires prolonged acquisition times to achieve adequate signal-to-noise ratios (SNR), limiting its practicality for large-scale studies. For instance, Legrand et al. reported a macroscopic FT-IR scanning system in reflection mode (MA-rFT-IR) that integrates a conventional portable single-point FT-IR spectrometer with a motorized stage to generate chemical maps. However, because of the extended acquisition time per point combined with additional overhead for data saving and stage movement, scanning an area of 5.5 × 8 cm^2^ at a lateral resolution of 1 mm required up to 36 h.^11,12^ Furthermore, the reflection-mode geometry itself introduces complications. Depending on angles, surface roughness, and layering, the resulting spectra of rFT-IR can exhibit distortions caused by specular reflections and diffuse reflections, making interpretation highly challenging.^13,14^ Consequently, if the spectra are noisy or ambiguous, then critical chemical information can be lost.

To mitigate these issues, several studies have therefore proposed the combination of data processing strategies with MA-rFT-IR point-by-point mapping to extract and interpret chemical information. Sciutto et al. proposed an automated approach integrating principal component analysis (PCA) with density-based spatial clustering of applications with noise (DBSCAN) algorithm for data collected from bronze surfaces.^15^ Capobianco et al. took a different route by performing midlevel data fusion on the MA-rFT-IR data with other hyperspectral imaging (HSI) data sets, spanning from the visible to MIR range, then applying PCA and algorithms to automatically classify different pictorial materials.^16^ Pronti et al. applied the same MA-rFT-IR mapping setup as Capobianco et al., with different acquisition parameters, focusing on the assessment of cleaning treatments and restoration efficacy on paintings.^17^ Nevertheless, limited attention has been given to the development of appropriate denoising strategies that preserve diagnostically relevant band shape while enabling shorter acquisitions for portable reflection-mode mapping. This gap is particularly relevant for portable systems operating under low-scan conditions, where denoising strongly affects the interpretability of subtle spectral features and the performance of multivariate analysis.

Building upon these considerations, the present study offers a systematic and quantitative evaluation of spectral denoising methods designed for portable reflection-mode mapping. The approach integrates region specific evaluation metrics, band sensitive assessments, and subsequent multivariate unmixing to examine how denoising influences both spectral features and chemical interpretability. This framework aims to provide a more informed and reproducible basis for selecting denoising strategies in practical MA-rFT-IR imaging applications.

In this context, the proposed framework is implemented on the in-house integrated portable MA-rFT-IR mapping system developed by the Authors. By combining a portable FT-IR spectrometer with a precision motorized stage and optimized spectral denoising techniques, this integrated approach significantly expands the practical applicability of MA-rFT-IR mapping systems. The method aims to balance acquisition speed, cost, and chemical specificity in single-pixel MIR scanning systems, an approach slower than visible-near-infrared (NIR) imaging yet capable of providing essential fingerprint-region information not accessible in the NIR, enhancing the practicality of MIR diagnostics. Importantly, the proposed approach does not rely on intrinsically faster MA-rFT-IR imaging instrumentation compared to previously reported systems (e.g., Legrand et al.). Instead, it is designed to enhance spectral interpretability and the reliability of chemical mapping, even under reduced-scan acquisition conditions.

The proposed portable MA-rFT-IR mapping system collects data point-by-point across both the NIR and the MIR regions (5500–650 cm^–1^). To minimize acquisition time, the number of scans per measurement is intentionally reduced, resulting in inherently noisy spectra. A comprehensive data processing workflow was therefore designed to systematically test multiple denoising methods, including (i) single-method approaches such as Savitzky–Golay filtering, wavelet denoising, and PCA-based denoising, and (ii) combinations of PCA-based denoising with either wavelet or Savitzky–Golay filtering. The performance of each method was evaluated through arccosine-based similarity (ACOS), derivative arccosine (dACOS), and relative derivative-based root-mean-square error (dRMSE).

Selection of candidate denoising methods was informed by FT-IR imaging benchmarks that document the strengths and limitations of common filters and multivariate denoising.^18–20^ These insights were adapted to the constraints of the portable system and reflection geometry.

The effectiveness of the strategy was validated using a varnished multilayer painting mock-up. After denoising through PCA-based and wavelet methods with optimized parameters, multivariate curve resolution–alternating least-squares (MCR-ALS) was used to interpret the complex reflectance profiles. The results were compared with those obtained before denoising to assess gains relative to conventional processing.

The present study frames denoising for portable MA-rFT-IR mapping under low-scan acquisition as an application-specific selection problem, using region-of-interest (ROI)-wise metrics that include a band-shape indicator (dACOS) alongside global similarity (ACOS) and high-frequency noise (dRMSE). Together, the cost-effective acquisition hardware and targeted band-aware denoising enable reliable chemical mapping at reduced scan counts, supporting more sustainable field practices.

## 2 Experimental Section

### 2.1 MA-rFT-IR Mapping System

The setup integrated a portable FT-IR spectrometer with a motorized stage to perform a macroscopic reflection-mode mapping (Figure 1). The core spectrometer is a commercial Agilent Cary 630 FT-IR benchtop model (Agilent Technologies, USA) equipped with the Madatec MadaIR noncontact external reflection accessory (Madatec S.r.l., Italy). The MadaIR accessory enables noncontact measurements based on specular/diffuse reflectance and can be installed on existing Cary 630 units while maintaining the original instrument specifications. The system operates in 5500 to 650 cm^–1^ at a 4 cm^–1^ spectral resolution. The focal distance to the sample surface is fixed at 22 mm with a spot size of approximately 2.0 mm.

**Figure 1:**
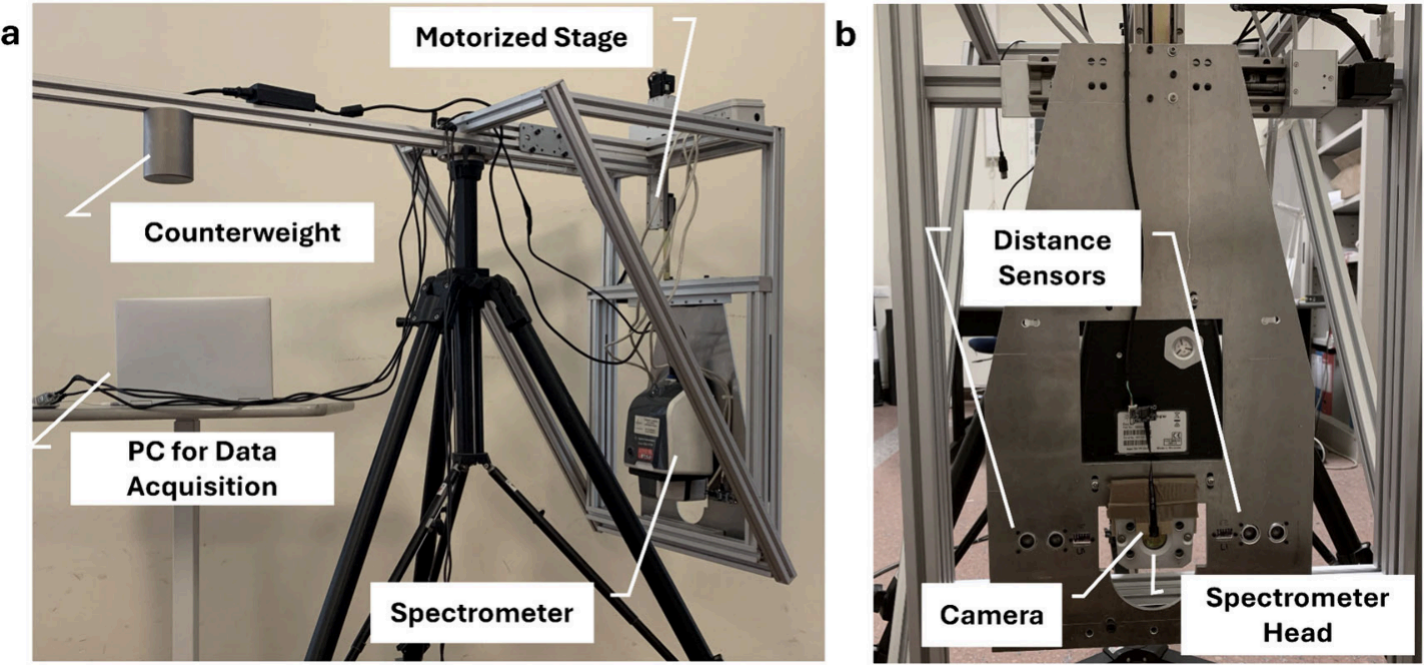
In-house integrated MA-rFT-IR mapping system: (a) lateral view shows the assembly of the motorized stage with the spectrometer; (b) front view shows the spectrometer mounted on the aluminum frame.

For two-dimensional mapping, the spectrometer is mounted on an aluminum frame equipped with precision motorized axes with a travel range of 150 mm per axis (model: CXN50150-S2NN-ND-P1, GMT Europe GmbH, Germany). These axes position the spectrometer in the *X*–*Y* plane, with user defining parameters such as step size, scanning speed, and ROI. Two distance sensors, time-of-flight (AZDelivery VL53L0X) and ultrasonic (AZDelivery HC-SR04), monitor the gap between the spectrometer and the sample, ensuring consistent focal alignment before and during the scan.

Spectral acquisition is managed through MicroLab PC software, while a custom MATLAB code (R2022b, The MathWorks, Inc., USA) controls stage movement and synchronizes acquisition. Since direct software integration is not available, an automated mouse-control routine launches each new measurement. The system collects data in a bidirectional boustrophedonic pattern, moving horizontally across the target area and then gradually down to the next line.

In this configuration, chemical maps are generated in a point-by-point (whisk-broom) scanning pattern using a single-element detector, with each pixel corresponding to one individual FT-IR spectrum acquired with a specified number of scans.

### 2.2 Data Acquisition

A three-layer painting mock-up (inspired by Botticelli’s *The Birth of Venus*) was used to evaluate the instrument performance and to collect reference data sets. Dammar varnish was applied only to the left half of the mock-up as the uppermost layer, while the right half was unvarnished. Additional details about the mock-up’s materials, stratigraphy, and preparation methods are provided in the Supporting Information (SI, Figure S1).

A 72 mm × 74 mm area on the mock-up was selected for MA-rFT-IR mapping, covering most of the *Venus* face (Figure 2). The mapping was collected with 32 scans, using a 2 mm step and a pixel size of 2 mm, at a movement speed of 10000 pulses per minute (ppm) with a focal distance of 22 mm from the mock-up surface. After acquisition, each spectrum was automatically saved and subsequently rearranged into a data tensor with dimensions of 37 × 38 pixels × 2603 spectral variables. The total acquisition time is approximately 11 h.

**Figure 2:**
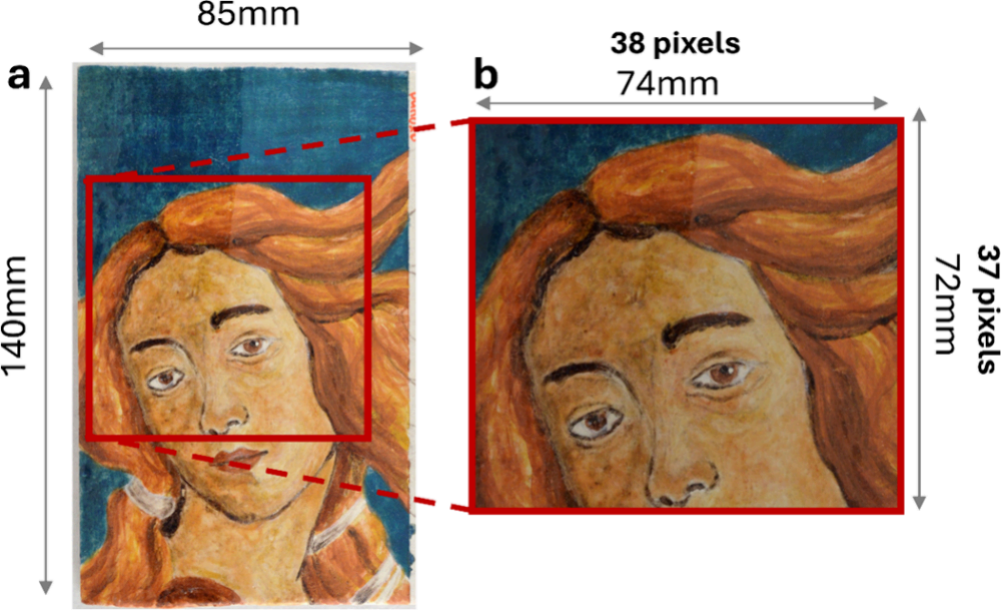
(a) Mock-up painting “Venus” with area of analysis highlighted in red; (b) enlarged view of the analyzed area showing detailed measurements.

To assess denoising efficiency, single-point data acquisition was conducted independently using the Agilent Cary 630 FT-IR spectrometer (5500–650 cm^–1^ with a resolution of 4 cm^–1^). Each reference spectrum was obtained with 1024 scans for enhancing SNR. Spectra were collected from both varnished and unvarnished areas within the blue background area of the *Venus* mock-up.

MATLAB was used for data rearrangement and subsequent data processing.

### 2.3 Spectral Denoising Methods

Prior to data processing, the reflectance spectra expressed in percent (%*R*) were converted into the pseudoabsorbance scale (*A* ^′^) using the equation A′=−log10(R100) .

Three denoising methods, Savitzky-Golay smoothing and wavelet- and PCA-based denoising, were tested, both individually and in combination, aiming to find the most suitable denoising method for the MA-rFT-IR map.

The specific denoising methods and their parameter configurations are detailed below:

1) Savitzky-Golay (SG) smoothing filter: The SG smoothing uses a polynomial fit within a moving window to reduce high-frequency noise.^21^ Two polynomial orders (2 and 3) were tested with odd-number window sizes ranging from 3 to 29.
2) Wavelet-based denoising: Wavelet denoising reduced noise by decomposing the spectral signal into components that present multiple frequency levels (according to the variation of signal) using wavelet functions. This multilevel decomposition enables the separation of noise, typically associated with high-frequency components, from spectral information. Thus, in the high-frequency bands, wavelet denoising applies a thresholding approach: values below a certain threshold (noise) are reduced or removed, while stronger components (real signal) are retained. Afterward, the signal is reconstructed, preserving key spectral features while minimizing distortion. Different wavelet families were tested based on their reported effectiveness in IR spectral denoising, particularly for accommodating both broad absorptions and spectral features.^18^ The following wavelet families and parameters were tested to optimize performance:
  - Coiflets (coif): wavelet orders 1–5; decomposition levels 1–3
  - Daubechies (db): wavelet orders 1–20; decomposition levels 1–3
  - Symlets (sym): wavelet orders 1–20; decomposition levels 1–3
    For all tests, a soft thresholding approach was applied with ‘sqtwolog’ universal threshold, which corresponds to $\sqrt{2\ln\left(n\right)}$ , where *n* is the number of data points in the spectrum, as proposed by Donoho and Johnstone.^22^ This threshold is scaled by an estimate of the noise standard deviation, which was computed as the median absolute deviation (MAD) of the wavelet coefficients at the first decomposition level, divided by 0.6745, following the method suggested in.^22^ The scaled threshold was then applied to the wavelet coefficients to reduce noise while preserving key spectral features.

3) PCA-based denoising (inverse PCA): Spectral data sets were subjected to low-rank reconstruction based on PCA. This approach, also known as abstract factor analysis (AFA)^23^ and referred to here as “inverse PCA” to distinguish it from exploratory PCA models, involves decomposing the spectra into principal components and reconstructing them by retaining only the most significant ones. The optimal number of components, tested from 1 to 50, was determined using two complementary evaluation metrics: the arccosine-based similarity (ACOS) and the derivative-based root-mean-square error (dRMSE).
4) Inverse PCA in combination with wavelet denoising: After initial application of PCA (using the most suitable number of principal components determined by evaluation metrics), spectra were then processed with wavelet denoising, where all wavelet families, order, and decomposition level parameters were tested as described above.
5) Inverse PCA in combination with Savitzky-Golay smoothing filter: Subsequent to the application of inverse PCA step, spectra were smoothed via the SG filtering with the polynomial order and window size described previously in step 1.

### 2.4 Spectral Denoising Evaluation

The denoising performance of the proposed approaches was evaluated using two complementary metrics: the arccosine-based similarity (ACOS) and the relative derivative-based root-mean-square error (dRMSE).

ACOS measures angular similarity, quantifying the global spectral shape agreement between the evaluated spectrum *Ŷ* and the reference spectrum *Y*_*ref*_.^24^ The cosine of the angle θ between the two vectors (spectra) was first computed via their dot product:

$$\cos\theta =\frac{\underset{i}{\sum }{Ŷ}_{i}Y_{ref,i}}{∥Ŷ∥∥Y_{ref}∥}$$ 1

Then cosθ was then converted to a 0–1 similarity scale via

Acos(Ŷ,Yref)=1−arccos(cos⁡θ)π2 2

An ACOS value of 1 indicates identical spectral shapes (0° angle), while 0 corresponds to an angle of 90° (orthogonal spectra). Thus, higher ACOS values imply a closer overall spectral similarity.

Due to the fact that the full-spectrum ACOS can be less sensitive to the fine structure alterations, which affect only a narrow set of variables, ACOS was additionally computed on first-derivative spectra and restricted to a diagnostic window. This derivative ACOS (dACOS) was evaluated in the 4260–4245 cm^–1^ region to probe the split band, attributed to the OH-stretching mode coupled with two distinct carbonate vibrations.^25^ The derivative emphasizes local band-shape changes and was used to monitor over smoothing, whereby merging the azurite split peak into a single band reduces the dACOS value, whereas preservation of the splitting maintains a higher dACOS.

To assess how effectively high-frequency noise is reduced, the relative dRMSE was used. By taking the first derivative of spectra, rapid variations are accentuated, making dRMSE particularly sensitive to high-frequency fluctuations.^26^

Formally, the first derivatives of both *Ŷ* and *Y*_*ref*_ were first computed, denoted ∇*Ŷ* and ∇*Y*_*ref*_ respectively. The relative dRMSE was then defined by

$$dRMSE\left(Ŷ,Y_{ref}\right)=\sqrt{\frac{\sum {\left(\nabla Ŷ-\nabla Y_{ref}\right)}^{2}}{\sum {\left(\nabla Y_{ref}\right)}^{2}}}$$ 3

A lower dRMSE indicates that ∇*Ŷ* is closer to ∇*Y*_*ref*_, reflecting that the high-frequency noise has been effectively reduced.

These two metrics were used for both individual and combination methods to quantify the improvement achieved through noise reduction and serve as the basis for assessing the performance of each method and parameter setting.

An estimation of the SNR in reflectance units was also computed. For each ROI, the signal was defined as the mean reflectance of the evaluated spectrum (raw or denoised) within the corresponding spectral range,

$$S_{raw}=mean\left(R_{raw\_ROI}\right)$$ 4

or

$$S_{denoised}=mean\left(R_{denoised\_ROI}\right)$$ 5

and the noise was estimated as the standard deviation of the difference between the evaluated spectrum (raw or denoised) and the corresponding 1024-scan reference spectrum,

$$\sigma_{noise\_raw}=std\left(R_{raw\_ROI}-R_{ref\_ROI}\right)$$ 6

or

$$\sigma_{noise\_denoised}=std\left(R_{denoised\_ROI}-R_{ref\_ROI}\right)$$ 7

An effective SNR was then computed as *SNR* = *S*/σ_*noise*_ for both the raw and denoised conditions.^27,28^ The reported values correspond to the average SNR calculated over the ten randomly selected pixels within each area.

All denoising routines and metric evaluations were implemented in MATLAB.

### 2.5 Multivariate Spectral Unmixing for Denoised Data

To further explore the feature complexity of the reflectance profiles, MCR-ALS was applied to the denoised data tensor. In the present study, all MCR-ALS analyses were carried out using MCR-ALS GUI toolbox for MATLAB by Tauler et al.^29,30^ Before the analysis, a standard normal variate (SNV) transformation was applied to the spectra to correct the global intensity effect.^31^

The optimal number of components was determined by using singular value decomposition (SVD). Seven components were selected for the model. Following this, initial estimates for the spectral profiles were generated using the toolbox’s purest variable detection method.^30,32,33^

The data tensor was processed as two submatrices (MIR and NIR) in the *Definition of the 3-way data set* window, using the “Row-wise augmented data matrix (S direction)” option. The ALS optimization was then performed with non-negativity constraints to the concentration profiles, while the spectral profiles were left unconstrained. Euclidean (vector) normalization was applied at every ALS cycle, removing overall intensity differences. The optimization was considered converged when the change in the standard deviation of the residuals between consecutive iterations was less than 1%.

## 3 Results and Discussion

### 3.1 Instrument Performance (Raw Data)

The performance of portable MA-rFT-IR mapping was carried out with 32 scans per point, resulting in a map measuring 74 × 72 mm on the *Venus* face of the painting reconstruction, using a 2 mm step size. The scanned area included both varnished and unvarnished regions (Figure 2). 1024-scan spectra from the same regions were acquired to provide instrument-based reference for quantitative comparison.

Two 32-scan spectra from the blue azurite background, one from the varnished area and another from unvarnished, were presented in Figure 3, and compared with the corresponding 1024-scan reference spectrum acquired from the same location.

**Figure 3:**
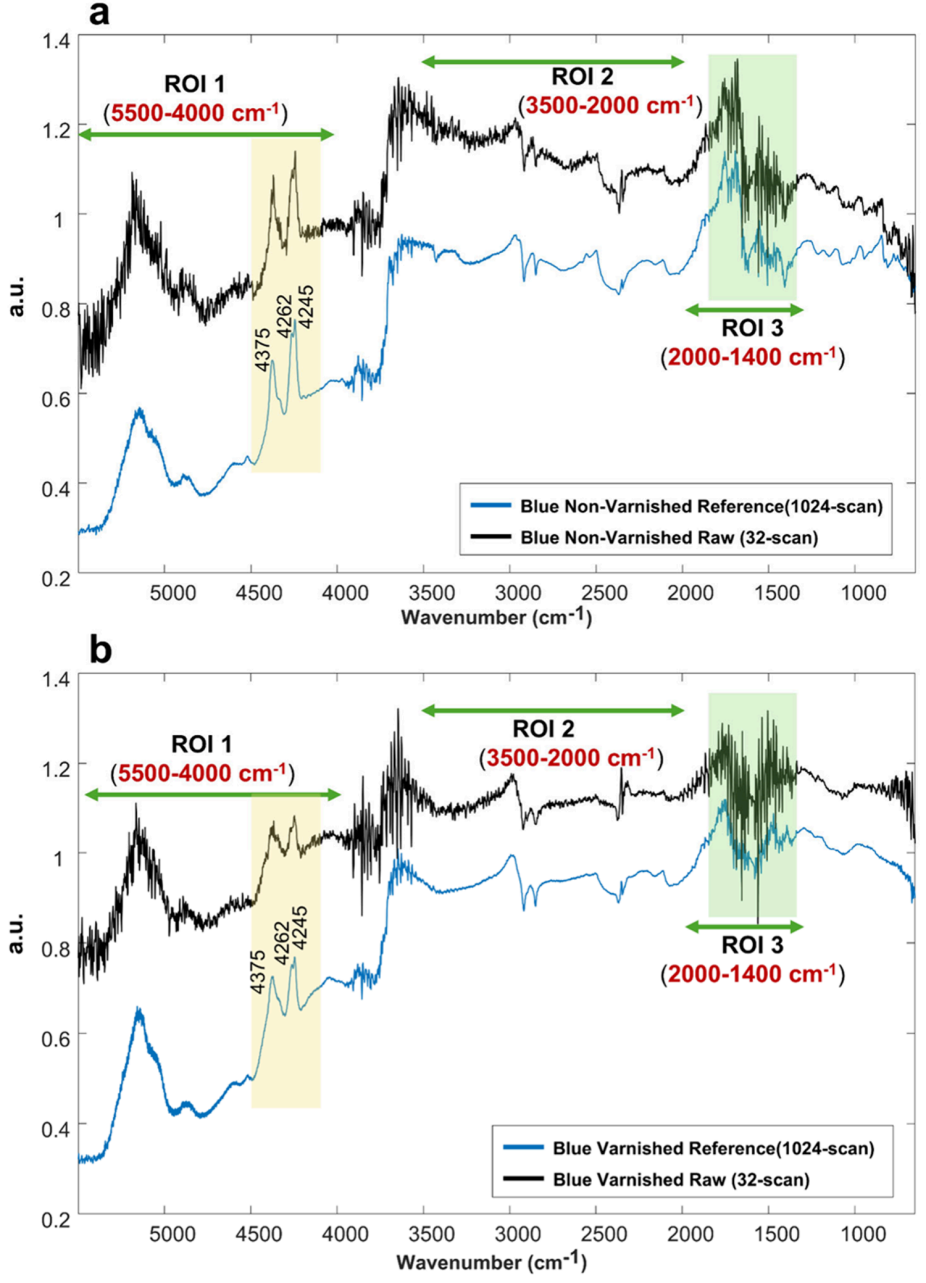
Comparison between the raw 32-scan spectrum (black) and the 1024-scan reference spectrum (blue) extracted/acquired from the nonvarnished and varnished blue areas of the mock-up painting. The green arrows indicate the three spectral ROIs. The yellow-shaded area highlights the combination and overtone bands of azurite in the NIR region, while the green-shaded area corresponds to the amide bands of the binder, which are partially obscured by atmospheric water vapor interference. (a) Raw spectrum extract from the blue nonvarnished area at point (3,32) compared with reference spectrum. (b) Raw spectrum extract from the blue varnished area at point (1,6) compared with reference spectrum.

Spectra acquired in reflection mode exhibited the typical features of this geometry, including inverted and derivative-like shapes as well as the presence of overtone and combination bands. In Figure 3, beyond the fundamental OH-stretching vibrations, azurite showed the combination ν+δ (OH) and overtone 3ν_3_ doublet at 4375 and 4245 cm^–1^ (highlighted in yellow in Figure 3).^13,34^ It is worth noting, in both raw and reference spectra, the lower-frequency member of this doublet, which is located at approximately 4245 cm^–1^, splits into two distinct subpeaks around 4260 cm^–1^ and 4245 cm^–1^ (highlighted in yellow in Figure 3). This splitting was caused by the combination of the main OH stretching vibration with two distinct carbonate group vibrations in azurite.^25^ Meanwhile, since both specular and diffuse reflection contributed to the signal, band intensities and baselines fluctuated across the entire wavenumber range under the influence of scattering effects. In the 32-scan spectra, binder signals were masked by strong interference related to atmospheric water-vapor rotational transitions. The interference introduced broad fluctuations in the 2000 – 1400 cm^–1^ region (highlighted in green in Figure 3), and partially hindered diagnostically relevant features, specifically the bands associate with proteinaceous binder around 1680 cm^–1^ (amide I) and 1560 cm^–1^ (amide II).^35^

Furthermore, the reduced number of scans introduced heteroscedastic spectral noise, arising from both random high-frequency fluctuations and reflectance-related baseline instabilities inherent to point-by-point mapping.^36^ These combination effects collectively complicate the detection and interpretation of subtle spectral features and underscore the need for effective denoising strategies. Due to difficulties in reproducing this type of noise through artificial simulations, the proposed method was designed under realistic acquisition conditions, operating directly on spectra containing real, instrument-, and sample-derived variability.

### 3.2 Denoising Methods Evaluation and Selection

#### 3.2.1 Evaluation Metrics

To evaluate the performance of the denoising methods, in terms of band shape preservation and reduction of relative intensity high-frequency fluctuations (noise), three spectral ROIs were defined (indicated in Figure 3). ROI 1 (5500–4000 cm^–1^) and ROI 2 (3500–2000 cm^–1^) include intense vibrational features attributed to pigments, binders, and varnishes, whereas ROI 3 (2000–1400 cm^–1^) corresponds to a spectral region often influenced by water vapor absorption, scattering effects, and baseline distortions.

The denoising test and evaluation workflow is illustrated in Figure 4 and consists of three main steps. First, the entire 32-scan raw data tensor was processed by different denoising methods, which were tested individually and in combination. Second, for each denoised data tensor, ten pixels located near the reference acquisition point were randomly selected from two blue areas (varnished and unvarnished). Third, the extracted denoised spectra from these pixels were compared with the corresponding 1024-scan measured reference spectrum from the same area and calculate the evaluation metrics.

**Figure 4:**
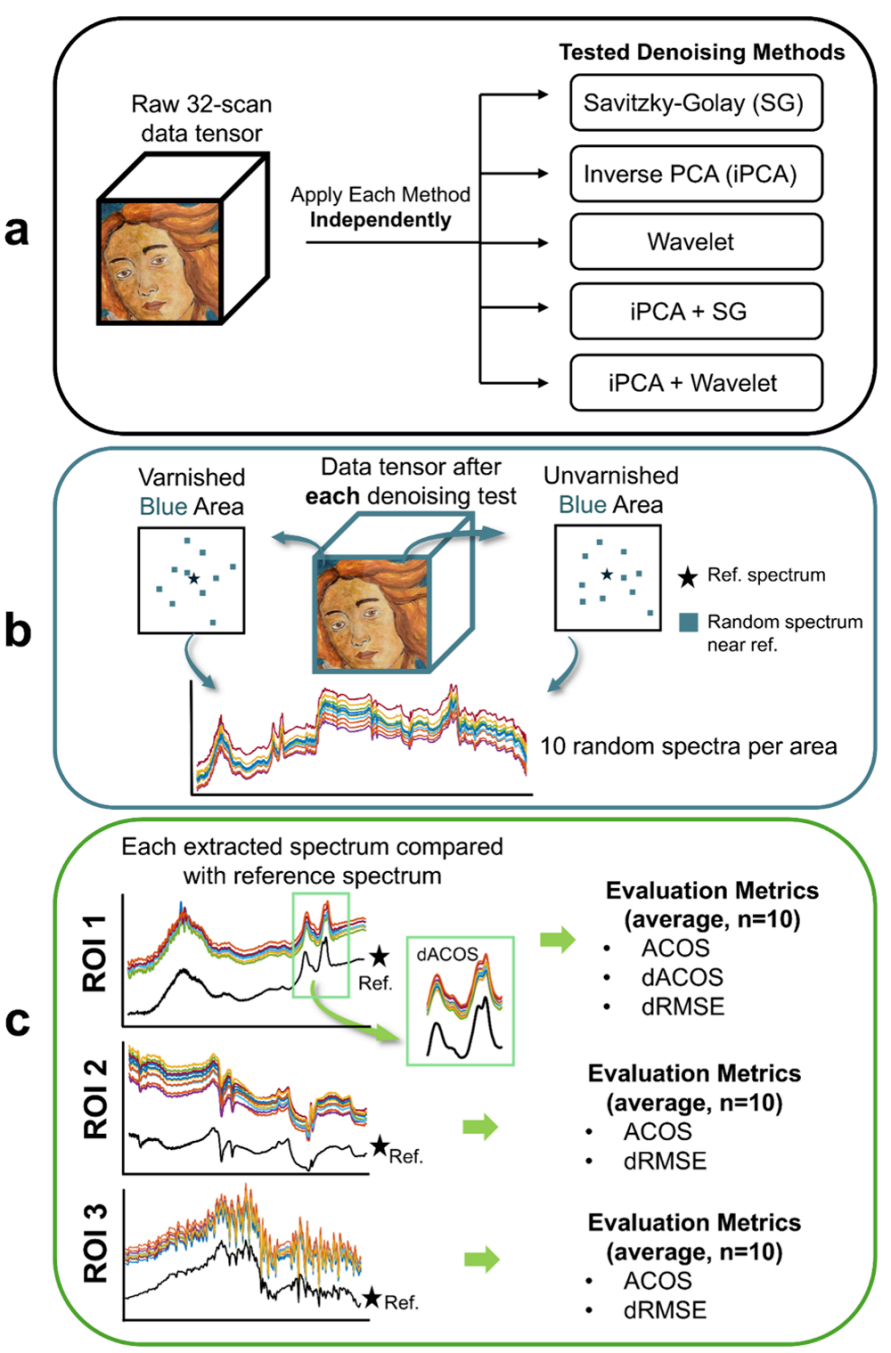
Schematic workflow for the evaluation of denoising methods. (a) Raw 32-scan data tensor was processed independently by different denoising methods with different parameters. (b) For each resulting denoised data tensor, ten pixels were randomly selected close to the reference point from unvarnished and varnished blue areas, and their spectra were extracted. (c) The extracted denoised spectra were compared to the corresponding reference spectrum within ROIs. Performance was quantified using ACOS, dRMSE, and, for ROI 1 only, dACOS. The final metric values were reported as the average over the ten selected pixels (*n* = 10).

This comparison was performed using quantitative metrics calculated for each spectral ROI. Specially, ACOS and dRMSE were calculated for all three ROIs, and dACOS was calculated on first-derivative spectra in 4260–4245 cm^–1^ (only in ROI 1) to track the azurite split peak. To quantify improvement relative to the unprocessed data, the same per-ROI calculations were applied to the raw 32-scan spectra to establish initial values for each ROI. All values were summarized and reported as the mean over the ten pixels (n = 10).

ACOS quantifies and assesses the global spectral similarity to the 1024-scan reference across the ROI window. dACOS tracks the azurite split peak in the region 4260–4245 cm^–1^, which decreases if over smoothing merges the split. dRMSE reflects the reduction of noise. Higher ACOS and dACOS and lower dRMSE indicate better performance, and changes relative to the raw 32-scan condition were used to compare methods and select the final configuration.

ACOS values computed between the reference spectrum and raw data were globally high and similar across all three ROIs under both surface conditions:

- ROI 1:0.91 (nonvarnished)/0.91 (varnished);
- ROI 2:0.98 (nonvarnished)/0.99 (varnished);
- ROI 3:0.98 (nonvarnished)/0.97 (varnished).

These consistently high ACOS indicated that even with low scan numbers mapping data generated by the MA-rFT-IR system preserves the overall spectral agreement with the reference spectral profile.

By contrast, the high values of dRMSE revealed the presence of high-frequency noise, which may lead to the loss of subtle features:

- ROI 1:3.67 (nonvarnished)/3.11 (varnished);
- ROI 2:2.53 (nonvarnished)/1.63 (varnished);
- ROI 3:1.20 (nonvarnished)/1.82 (varnished).

It is important to note that although the dRMSE in ROI 3 is numerically lower than in the other two ROIs, this reflects the characteristics of that window rather than lower noise in the mapping data. In ROI 3 the reference spectrum itself shows reduced band contrast and residual atmospheric, water-vapor structure typical of reflection mode (Figure 3). A smaller dRMSE in ROI 3 therefore does not imply clearer diagnostic information. In fact, the binder amide bands (∼1680 and ∼1560 cm^–1^ (highlighted in green in Figure 3) are barely visible in the 32-scan spectra. To avoid this confounding effect, method evaluation and parameter selection were performed using ROIs 1 and 2. The selected method was then applied to ROI 3 with a minor parameter adjustment to account for its comparatively high noise.

The denoising methods were tested individually with different parameter settings (see 2.3. Spectral Denoising Methods for details). Afterward, the methods were compared and then applied in combination.

#### 3.2.2 Individual Denoising Methods

For SG smoothing, a clear trade-off emerged between peak preservation (dACOS) and noise reduction (dRMSE). In nonvarnished spectra, increasing the window size lowered dACOS from 0.85 (order 2, window 13) to 0.72 (order 2, window 29) while reducing dRMSE from 1.19 to 0.63. The varnished area showed the same trend. Lower dACOS corresponds to over smoothing that merges the azurite split peak. Higher dACOS retains the split but the residual noise becomes unacceptable (examples in Figure S2).

A similar trend is observed in wavelet-based denoising parameters. Among the three wavelet families tested (db, symlet, and coif), symlet provided the best overall performance. Within the symlet family, the decomposition level of 3 consistently produced dRMSE values lower than those of level 2 for each tested order (from 1 to 20). The minimum dRMSE (0.66) was achieved with symlet order 6 at level 3 for nonvarnished area, but reduced dACOS to 0.76. The same order at level 2 preserved the split better (dACOS 0.83) but with a higher dRMSE (1.74). Examples are shown in SI Figure S3.

Across both surface conditions (varnish and nonvarnished), inverse PCA obtained higher dACOS values than either SG or wavelet methods (averaging 0.90 in the nonvarnished and 0.82 in the varnished spectra), demonstrating better preservation of overall spectral structure. This preservation, however, was accompanied by comparatively elevated dRMSE values with respect to noise reduction obtained with the other methods. For instance, denoising with 38 principal components (PCs) maximized dACOS value to 0.92, but produced a high dRMSE of 1.99. At the opposite extreme, using only three PCs achieved the lowest dRMSE value at 1.10 but reduced dACOS to 0.77, as also visible in the spectra profiles (see Figure S4, Supporting Information). Moreover, it should be noted that the number of PCs is of critical importance given that a reduction in PCs directly results in the removal of information contained within the data. By observing the dRMSE values gradually increased with PC numbers, a distinct jump appeared at approximately 15 PCs for both nonvarnished and varnished in ROI 1 (Figure S5). Accordingly, 15 PCs can be considered as the optimal compromise for inverse PCA (dRMSE = 1.11, dACOS = 0.91), for the spectral feature preservation, with a partial noise reduction. Nevertheless, residual high-frequency noise remains evident in the 15PCs spectra (Figure S4b, Supporting Information).

The comparison with detail values of each method is summarized and reported in SI Table S1.

#### 3.2.3 Combination of Denoising Methods

None of the individual denoising methods provided a universally optimal trade-off between achieving sufficient noise reduction and preserving both global and local spectral preservation. Therefore, two-step combination denoising methods were adopted: inverse PCA with 15 PCs was applied first to recover the primary spectral profile, and addressing the remaining noise was subsequently tested by using SG smoothing and wavelet denoising as the secondary step.

All SG and wavelet parameters were retested on the data denoised by inverse PCA (15 PCs).

Both the combination, inverse PCA followed by SG or wavelet, achieved a maximum dACOS of 0.92 in nonvarnished and 0.83 in varnished spectra. The corresponding dRMSE values indicated slightly lower dRMSE for the combination of inverse PCA and wavelet denoising using order 16 at level 2 (0.57 for nonvarnished, 0.69 for varnished samples), which was therefore selected as the final denoising strategy and parameter set.

To provide a clear visual summary of denoising performance and to enable an objective comparison, the results of each method are presented in two plots. Figure 5 compares all tested methods with different parameters. Figure 5a shows a Pareto front plot for ROI 1, using values averaged over the varnished and nonvarnished areas in the (dRMSE, dACOS) space. Points toward the top-left indicate the optimal combination of lower noise (smaller dRMSE) and better split-peak preservation (larger dACOS). The black star marks the final selected denoising method: inverse PCA (15 PCs) combined with a wavelet (symlet 16, level 2). Figure 5b presents an overall ranking based on a composite score (equal-weight normalization of dACOS and dRMSE reduction). The ranking corroborates the Pareto selection, showing that inverse PCA with wavelet ranks first, followed by inverse PCA with SG smoothing, whereas single methods rank lower due to either over smoothing (SG/wavelet) or residual high-frequency noise (inverse PCA alone).

**Figure 5:**
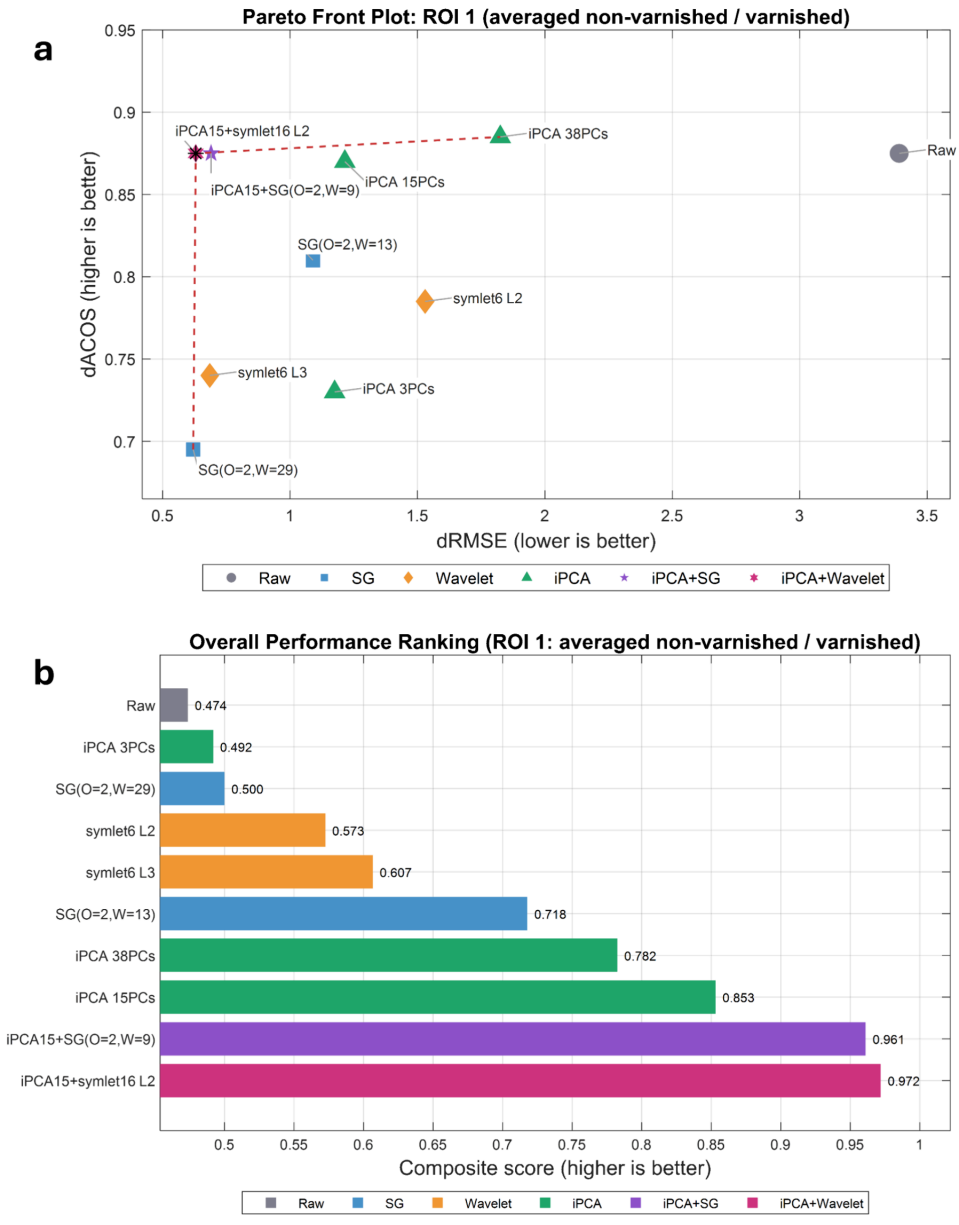
Evaluation and comparison of denoising methods with different parameters. (a) Pareto front in the (dRMSE, dACOS) space for ROI 1 (averaged across nonvarnished and varnished areas). Black star in the top left indicated the optimal solution and the final selected method: inverse PCA (15 PCs) with wavelet (symlet 16, L2). (b) Overall ranking is based on a composite score (equal-weight normalization of dACOS and dRMSE reduction); higher is better. The ranking is consistent with that of panel A.

All ROI 1 values correspond to the data plotted in Figure 5 for each method and parameter setting in both varnished and nonvarnished conditions, are compiled in Table S1 (SI).

### 3.3 Denoising Performance

The final denoising was applied on the tensor separately by region: ROI 1 and 2 were processed with inverse PCA (15 PCs) followed by symlet order 16 at level 2, whereas ROI 3 and another water-vapor–affected region underwent the same method but symlet order 16 at level 3 in the secondary wavelet smoothing. A direct comparison between the chosen inverse PCA + wavelet combination and the alternative inverse PCA + SG smoothing is provided in Figure S6 of the Supporting Information.

The performance with the final optimized parameters presented in Figure 6 is compared to the reference and raw spectra.

**Figure 6:**
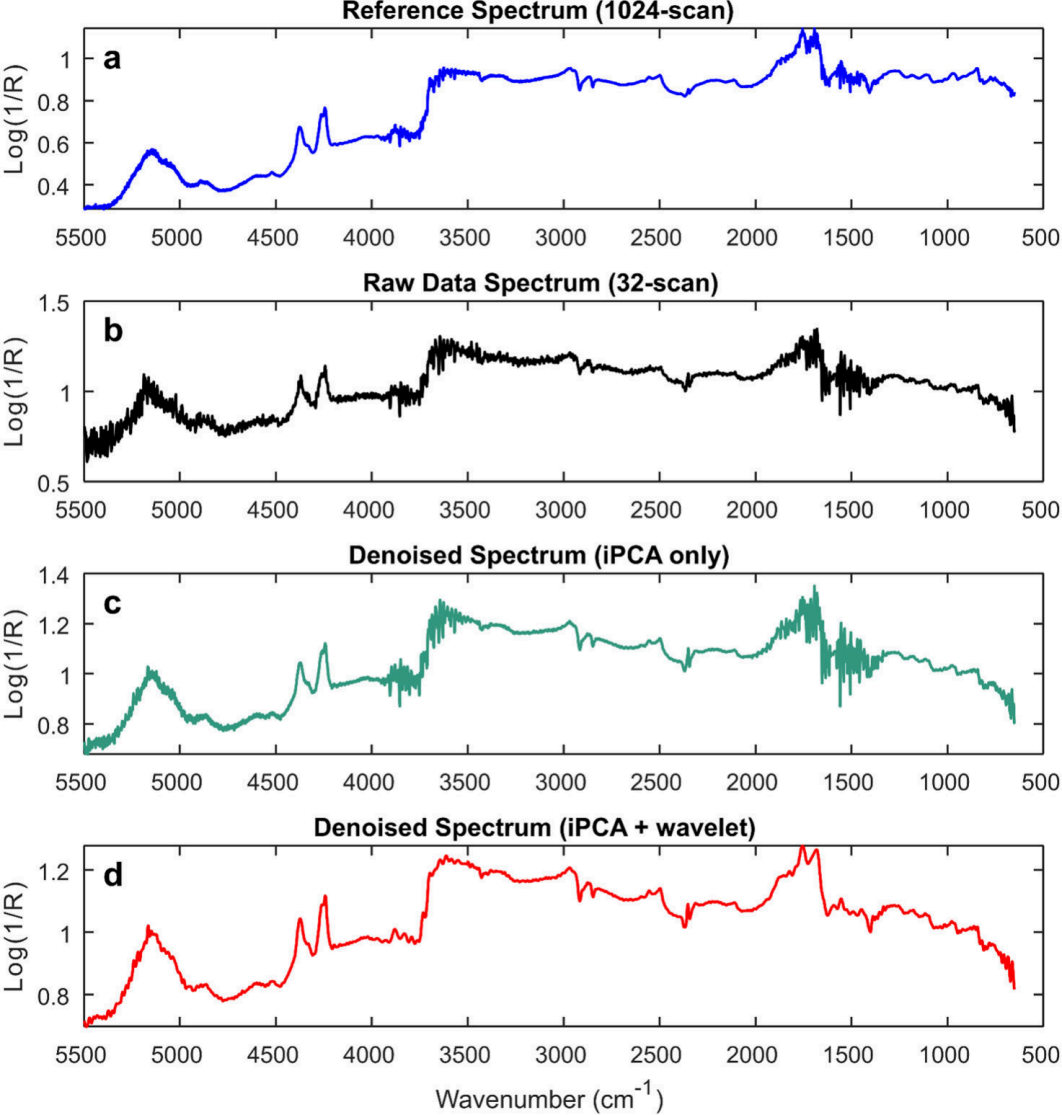
Effect of denoising through comparison of spectra from blue nonvarnish area: (a) reference spectrum collected with 1024 scans; (b) spectrum extracted at point (3, 32) from 32-scan mapping data; (c) spectrum in (b) denoised using inverse PCA (15 PCs); (d) spectrum in (b) denoised using inverse PCA (15 PCs) and wavelet (symlet16, level 3 for water-vapor regions and level 2 for the rest).

To quantify the improvement, SNR was computed in selected ROIs by dividing the mean ROI reflectance of the evaluated spectrum (raw or denoised) by the standard deviation of its residual relative to the corresponding 1024-scan reference. The denoising step increases the ROI-averaged SNR across all tested regions (Table 1).

**Table 1:** Average SNR Values for the Raw and Denoised (iPCA+Wavelet) Spectra Across the Three Rois, Computed over Ten Randomly Selected Pixels

Area		ROI 1	ROI 2	ROI 3
Nonvarnished	Raw	17.44	32.56	41.68
	Denoised	21.99	34.31	61.50
Varnished	Raw	12.30	83.76	23.1
	Denoised	14.34	112.51	42.90

Although individual spectral comparisons (Figure 6) highlight the effectiveness of the denoising procedure, it is also important to verify the performance across the complete data tensor. To that end, the entire data set was processed before and after denoising, with both sets subsequently undergoing SNV correction.

Figure 7 highlights one example of improvements by focusing on the amide II band at 1558 cm^–1^.^37^ Panels (a) and (c) were extracted from the same point (19,19) in the map but before and after denoising, revealing a cleaner profile and sharper features post-treatment. Panels (b) and (d) compare the distribution map of the 1558 cm^–1^ band before and after denoising, illustrating that the noise present in the untreated data obscures subtle signal variations, whereas the treated data result in a more coherent spatial distribution of the amide II feature. Thus, the band, related to the presence of proteinaceous binder, became clearly visible after the treatment in the painting area without varnish, demonstrating that minor but diagnostically relevant bands remain visible.

**Figure 7:**
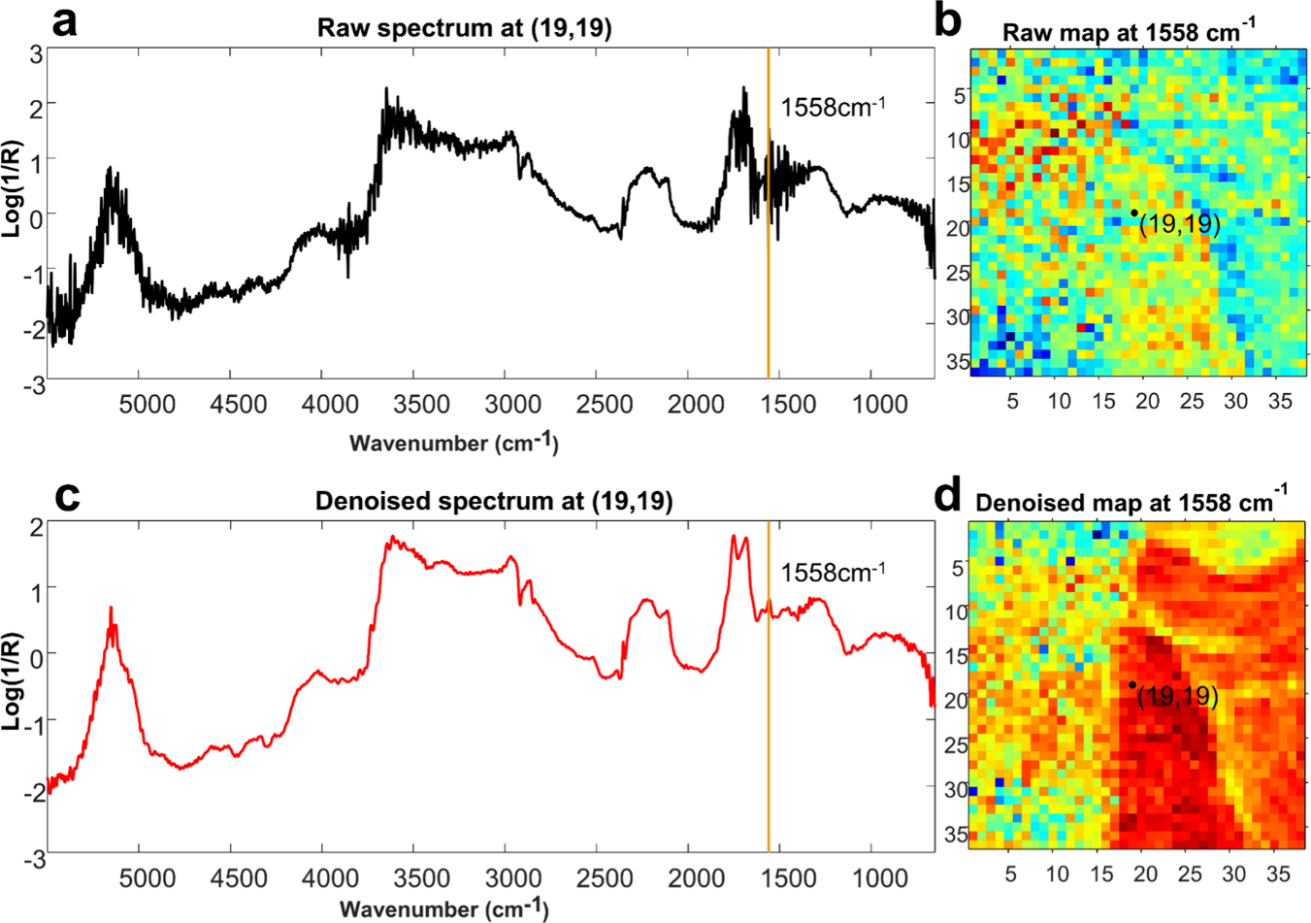
Comparison of the mapping data before and after denoising (with SNV in both cases): (a) spectrum extracted at point (19, 19) before denoising, highlighting the band at 1558 cm^–1^, related to the amide II band in the binder; (b) distribution map of the band at 1558 cm^–1^ before denoising; (c) spectrum extracted at point (19, 19) after denoising highlighting the band at 1558 cm^–1^, related to the amide II band in the binder; (d) distribution map of the band at 1558 cm^–1^ after denoising.

### 3.4 MCR-ALS Results: Spectral Unmixing and Interpretation

MCR-ALS was applied to decompose the denoised data tensor (after SNV) into resolved spectral profiles and concentration maps. The optimization converged after 110 iterations, explaining 99.8% of the total variance with a residual standard deviation of 0.041. These results indicate satisfactory reproduction of the experimental data and confirm the adequacy of the selected number of components and applied constraints. Although rotational ambiguity is inherent to MCR, allowing multiple mathematically equivalent solutions to reproduce the same data set, in this case the decomposition was found to be stable and reproducible. Repeated runs using the same data set, initialization, and constraint settings consistently produced identical concentration and spectral profiles. Therefore, the interpretation of the resolved components can be considered reliable, and subsequent discussion focuses on the chemical and spectral consistency of the extracted components.

Figure 8 presents the five key components with the corresponding distribution maps and resolved spectra.

**Figure 8:**
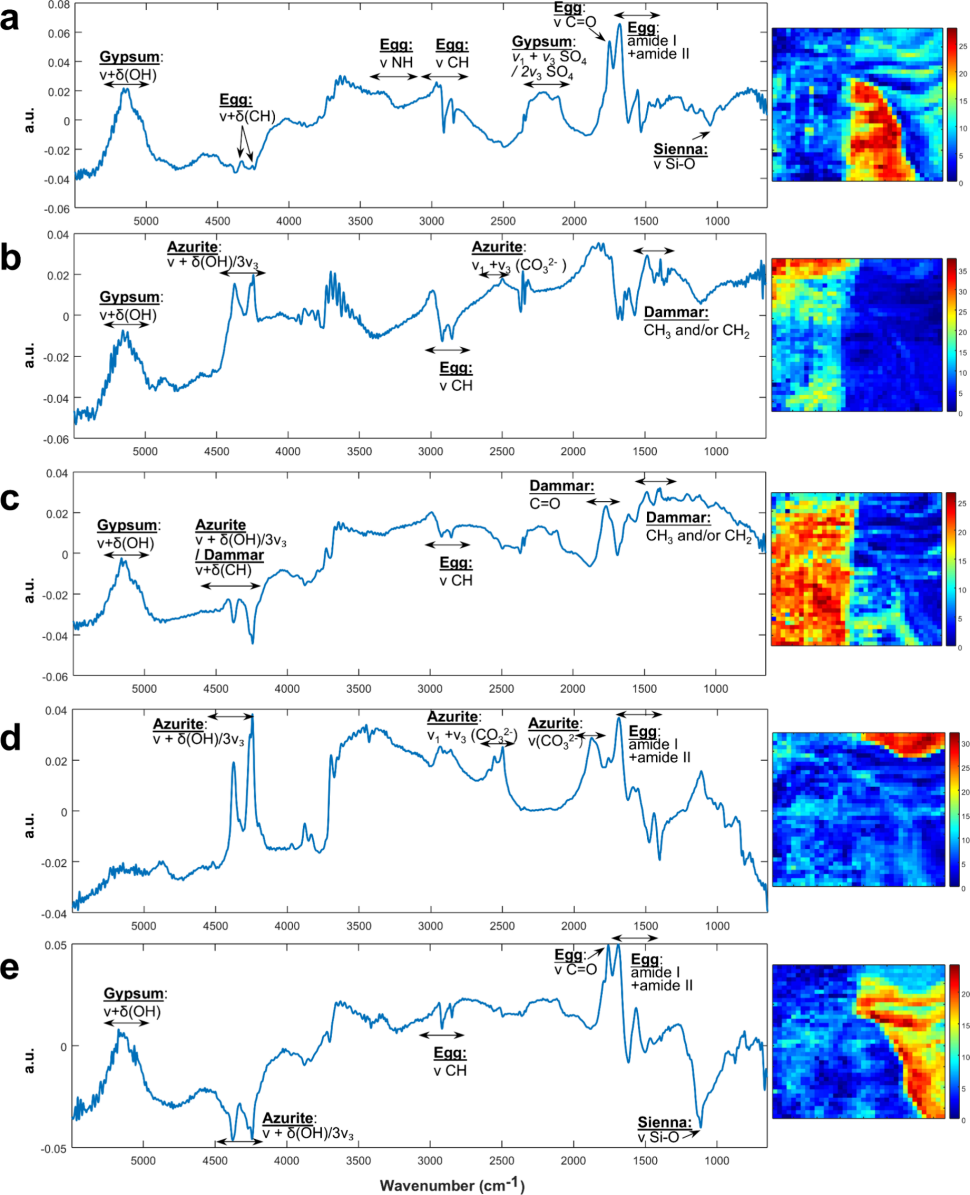
MCR-ALS results obtained from denoised data (after SNV transform) presenting the resolved spectral profiles (left) and component distribution maps (right) for five key components. Each distribution map indicates the relative concentration of the respective component (blue to red scale), while the spectra highlight characteristic spectral features. (a) Component 1: mainly represents the egg binder and gypsum constituting the ground layer, highlighted in the nonvarnished area. (b) Component 2: describes the varnished region, showing dammar features together with signals of azurite and egg. (c) Component 3: mainly dominated by dammar bands. (d) Component 4: corresponds to the unvarnished azurite region, showing characteristic bands of azurite and egg binder. (e) Component 5: identifies silicate-based earth pigments (Sienna) localized in the hair area.

The first component (Figure 8a) predominantly separated the egg binder and gypsum constituting the ground layer, which could be detected in both the NIR and MIR regions. The compositional distribution map highlighted this component in red, mainly in the nonvarnished half of the *Venus* face. The corresponding spectra (Figure 8a) revealed characteristic bands about 1680 and 1560 cm^–1^ for amide I and II, respectively, and at 1750 cm^–1^, attributed to ester C = O stretching of lipids, which are typical for egg binder.^35^ Additionally, bands around 2800–3000 cm^–1^ correspond to C–H stretching modes, around 3300 cm^–1^ for NH stretching of amide groups, and those at 4260 and 4333 cm^–1^ are ascribed to ν+δ (CH).^35,37^ Bands observed at 2115 and 2223 cm^–1^ are indicative of overtone and combination bands of SO_4_^2–^ groups, while the absorption around 5150 cm^–1^ with the shoulder about 5058 cm^–1^ can be attributed to the combination of stretching and bending OH in gypsum.^37,38^ Furthermore, band at around 1100 cm^–1^ indicates the presence of silicate-based pigment (Sienna).^35^

The second and third components (Figure 8b and c) mainly describe the varnished area. Especially the profile of the third component (Figure 8c) exhibited organic spectral features of dammar with bands at 1392 and 1483 cm^–1^ ascribed to CH_3_ and/or CH_2_ bending modes. Band at 1768 cm^–1^ and shoulder around 1735 cm^–1^ can be attributed to different carbonyl groups present in dammar varnish.^39^ Since the NIR radiation penetrates through the superficial layer, signals of pigments can also be observed in such components. Particularly, the NIR bands of highly absorbing pigment azurite at 4245 and 4374 cm^–1^ are evident. Interesting to note is that the bands of azurite are inverted in component three (Figure 8c), an indication that the MCR optimization trends to enhance the positive contribution of the varnish rather than azurite in this spectral region.

The fourth component clearly maps the area characterized by the presence of egg and azurite in the unvarnished area (Figure 8d). The compositional distribution map showed this component as red in the nonvarnished azurite regions. The spectrum (Figure 8d) exhibited characteristic band of azurite around 1880 cm^–1^ ascribed to ν_1_+ν_4_ (CO_3_^2–^) vibration, and at 2497, 2560, and 2600 cm^–1^ for ν_1_+ν_3_ (CO_3_^2–^).^13^ Notably sharp and intense bands at 4245 and 4374 cm^–1^ are identified as combination ν + δ (OH) and overtone 3ν_3_(CO_3_^2–^) in the NIR region.^13^ This component also contains spectral features of egg binder with bands at 1680 and 1558 cm^–1^ for amide I and II, respectively, and 1757 cm^–1^ for ester C = O.^37^

The fifth component (Figure 8e) additionally found the silicate-based earth pigments (Sienna) used in the hair area by presenting the characteristic band around 1110 cm^–1^ of Si–O antisymmetric stretching.^35^

A complete summary of all band assignments is provided in Table S2 of the Supporting Information.

To assess the impact of denoising on multivariate analysis, MCR-ALS was also performed on the raw SNV-corrected data (Figure S7, Supporting Information). Although the spatial distribution maps of key materials, such as silicate-based and azurite pigments, remained largely consistent, the raw resolved spectra exhibited significant high-frequency noise, particularly in regions affected by water vapor. As previously discussed, these spectral regions contain important diagnostic features, especially for identifying the egg-based binder, which remains difficult to interpret due to the residual noise.

## 4 Conclusions

The present research demonstrated that a targeted data-driven workflow can substantially improve the interpretability of the portable MA-rFT-IR mapping data acquired under low-scan condition. By quantitatively integrating noise reduction metrics (dRMSE) with spectral shape preservation indices (dACOS), the proposed approach showed that the combination of inverse PCA and wavelet-based denoising enhanced spectral interpretability while retaining the subtle diagnostic features. The resulting improvement in spectral clarity and feature preservation was directly reflected in the increased stability and readability of the MCR-ALS decomposition, underscoring the importance of appropriate preprocessing for analyzing multilayered paintings.

While the framework may be transferable to other point-scanning or single-pixel modalities with similar noise behavior, this study does not address the specific noise mechanisms of dispersive systems. Therefore, any extension to such modalities would require a dedicated investigation.

While the proposed data-driven workflow enhances the interpretability of low-scan portable MIR acquisitions, some methodological aspects should be considered to ensure its proper performances.

The denoising algorithms require the presence of a distinguishable spectral trace and cannot reconstruct diagnostic features when the initial SNR falls below the intrinsic detection capability of the sensor, and metrics such as dACOS naturally become less sensitive in regions dominated by weak or broad absorption features. In addition, because optical behavior and noise characteristics vary across the NIR–MIR range, denoising parameters are best optimized locally rather than through a single universal configuration. These considerations outline practical boundaries rather than limitations of the approach and highlight clear directions for future development, including adaptive parameter-selection strategies capable of adjusting denoising thresholds to local spectral characteristics and improving robustness across diverse surfaces. Finally, it is worth noting that the present validation was carried out on a controlled mock-up sample to enable a precise comparison of processed data and a reliable assessment of denoising effects; by knowing the expected spectral profiles and identifying unwanted distortions, the method was designed to be applicable to aged and complex artworks, whose in situ evaluation is a part of an ongoing research work.

## Supplementary Material



## Data Availability

Data supporting this study are available upon reasonable request from the Corresponding Author.

## Notes

Supplementary figures and tables provide additional details on the painting mock-up composition, comparison of denoising results obtained with Savitzky–Golay, wavelet, and inverse-PCA methods under different parameter setting, and the corresponding evaluation metrics, experimental wavenumbers and vibrational band assignments of the investigated materials, MCR-ALS results from the raw (nondenoised) data set for comparison (PDF)
