# Supplementary material for: Expanding the Capabilities of Portable Mapping in Macroscopic External Reflection FT-IR through a Targeted Data-Driven Spectral Enhancement and Denoising Strategy
Source: ACS Meas Sci Au. 2026 Feb 12;6(2):361–73. doi: 10.1021/acsmeasuresciau.5c00163 (PMC13087942; doi:10.1021/acsmeasuresciau.5c00163)
Supplement: Supplementary file 1 [file tg5c00163_si_001.pdf]

Supplementary Information

Expanding the capabilities of portable mapping in macroscopic external reflection FT-IR through a targeted data-driven spectral enhancement and denoising strategy

Zelan Li[a], Emilio Catelli[a], Jošt Stergar[b,c], Matija Milanič[b,c], Roberto Sáez-Hernández[d], Silvia Prati[a], Paolo Oliveri[e], Giorgia Sciutto[a*]

[a] Department of Chemistry "Giacomo Ciamician", University of Bologna, Via Guaccimanni, 42, Ravenna, 48121, Italy
[b] Faculty of Mathematics and Physics, University of Ljubljana, Ljubljana SI-1000, Slovenia
[c] Jožef Stefan Institute, Ljubljana SI-1000, Slovenia
[d] Department of Analytical Chemistry, Faculty of Pharmacy, University of Valencia, Dr. Moliner, 50, Burjassot, Valencia, 46100, Spain
[e] Department of Pharmacy (DIFAR), University of Genova, Genova, I-16148, Italy
[*] E-mail: giorgia.sciutto@unibo.it

Figure S1. Painting mock-up

The stratigraphy of the mock-up painting consisted of three distinct layers on the wooden support: 1) a preparation layer composed by gypsum and glue binder; 2) a pigment layer with egg binder, and 3) a layer of dammar varnish on the left half part of the mock-up. Four main pigments were used in the painting layer: azurite, burnt sienna, yellow ochre and zinc white. Pigments and binders were purchased from Zecchi (Florence, Italy) and Kremer Pigmente GmbH & Co. (Aichstetten, Germany). Details about each area and the corresponding components were reported in figure S1.

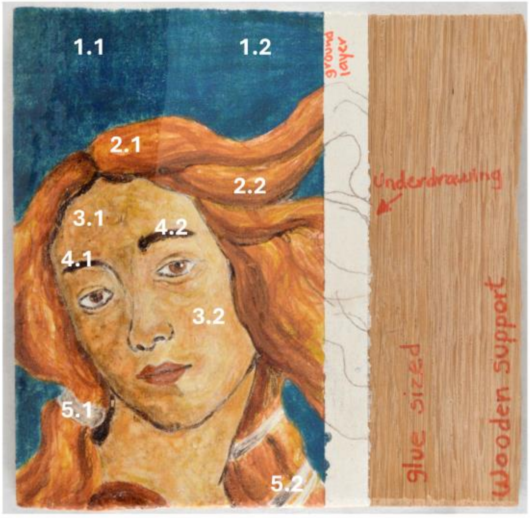

Area	Painting layer (with egg binder)	Varnish layer
1.1	Azurite	Dammar
1.2	Azurite	
2.1	Burnt sienna + yellow ochre	Dammar
2.2	Burnt sienna + yellow ochre	
3.1	Burnt sienna + zinc white	Dammar
3.2	Burnt sienna + zinc white	
4.1	Burnt sienna	Dammar
4.2	Burnt sienna	
5.1	Zinc white	Dammar
5.2	Zinc white	

*outlines, lips, nose and pupil of eyes: burnt sienna
** white of eyes: zinc white

Fig. S1: Mock-up painting "Venus" depicting numbered areas (left) and a table detailing the pigments and binders used in each respective area (right).

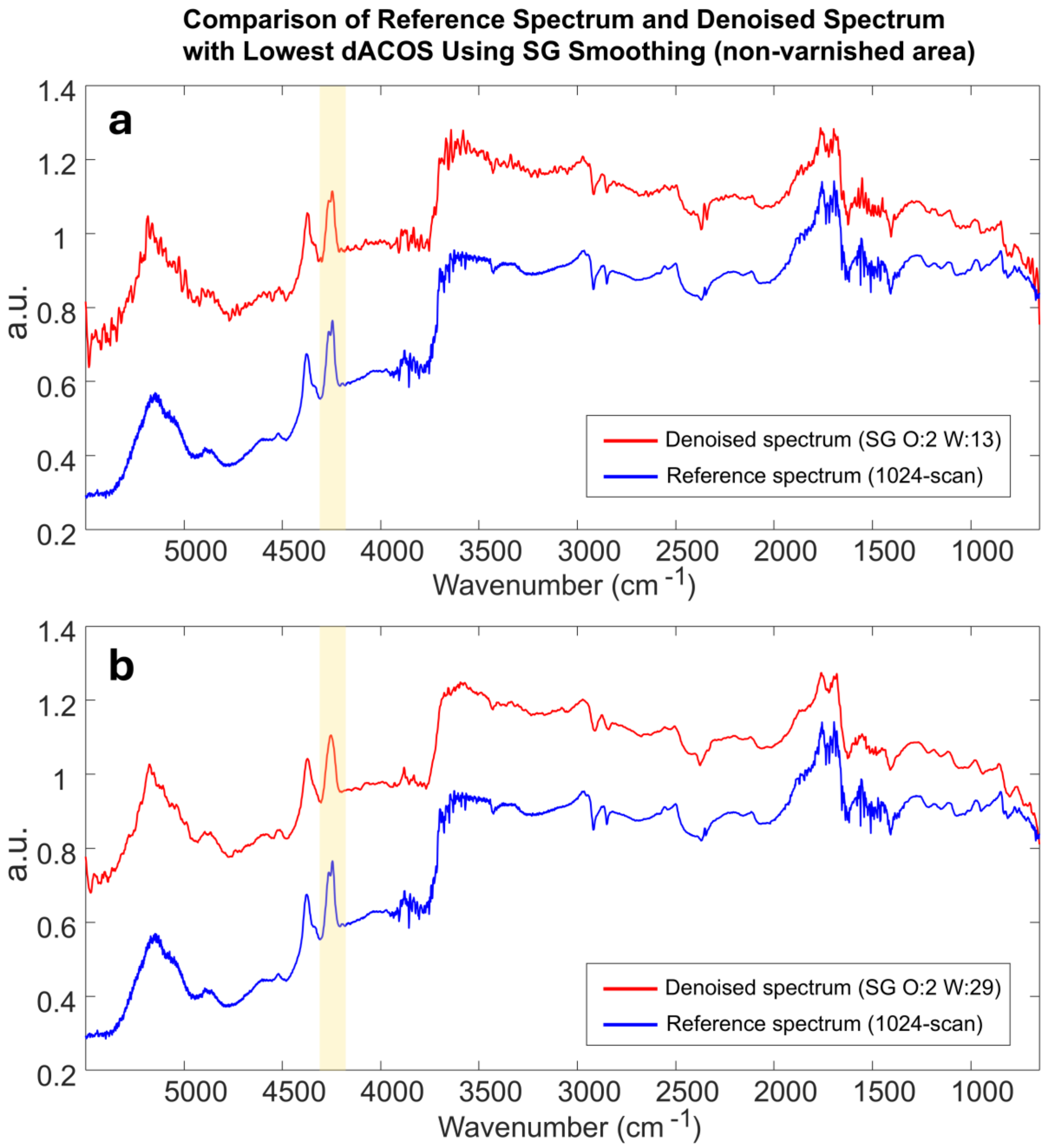

Fig. S2: Comparison of peak preservation and noise reduction in denoised spectra using SG smoothing from non-varnished blue area.

(a): Comparison of reference spectrum (blue) with denoised spectrum (red) using SG smoothing(Order 2, Window size 13), resulted in dACOS=0.85 (highest) and dRMSE=1.19. The preserved split-peaks of azurite highlighted in yellow.

(b): Comparison of reference spectrum (blue) with denoised spectrum (red) using SG smoothing(Order 2, Window size 29), resulted in dACOS=0.72 (lowest) and dRMSE=0. 63. The over-smoothed split-peaks of azurite highlighted in yellow.

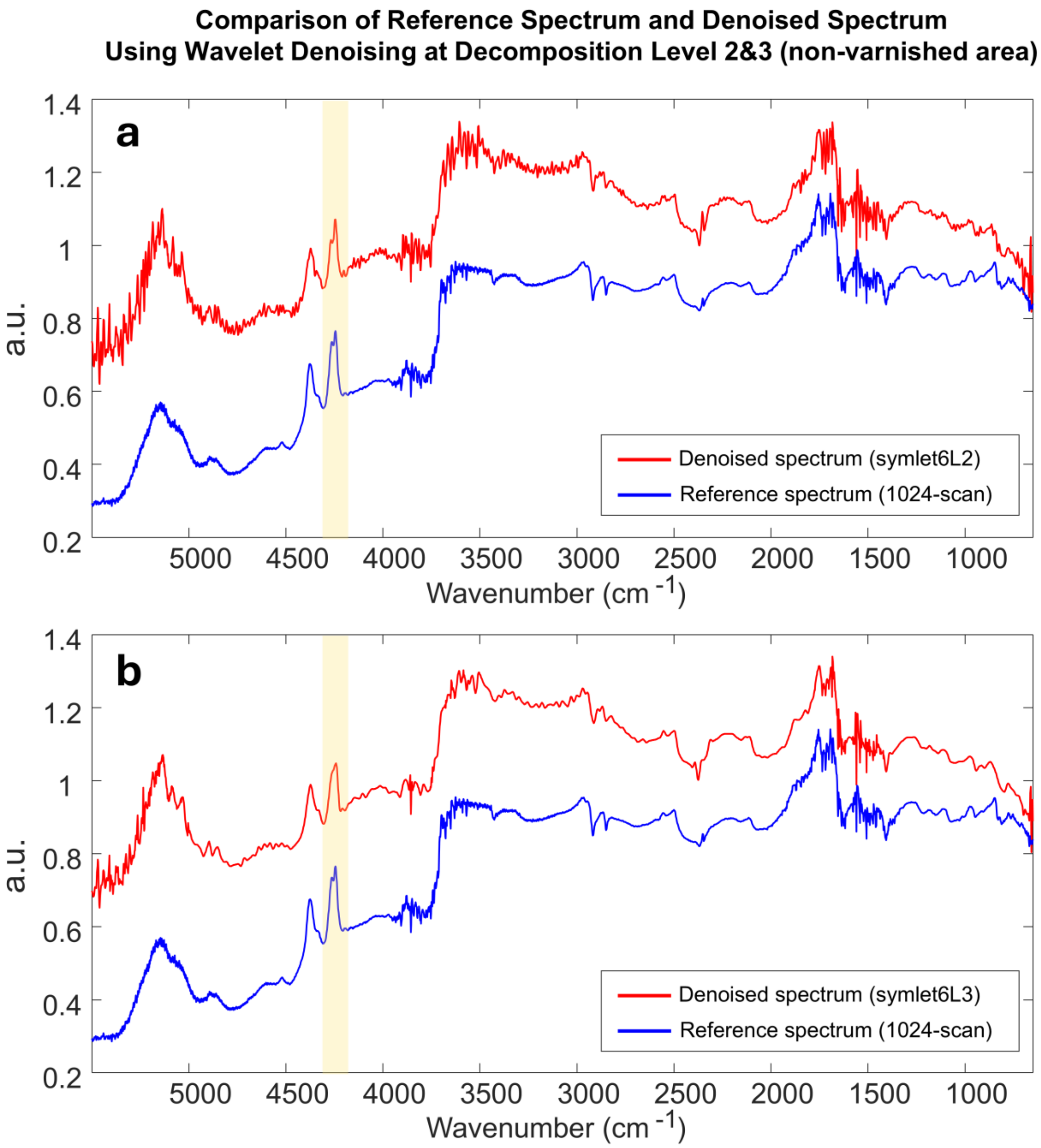

Fig. S3: Comparison of denoised spectra from non-varnished blue area using wavelet denoising at decomposition level 2 and 3.

(a): Comparison of reference spectrum (blue) with denoised spectrum (red) using wavelet (symlet 6, level 2), resulted in dACOS=0.83 and dRMSE=1.74. The preserved split-peaks of azurite highlighted in yellow.

(b): Comparison of reference spectrum (blue) with denoised spectrum (red) using wavelet (symlet 6, level 3), resulted in dACOS=0.76 and dRMSE=0. 66 (lowest). The over-smoothed split-peaks of azurite highlighted in yellow.

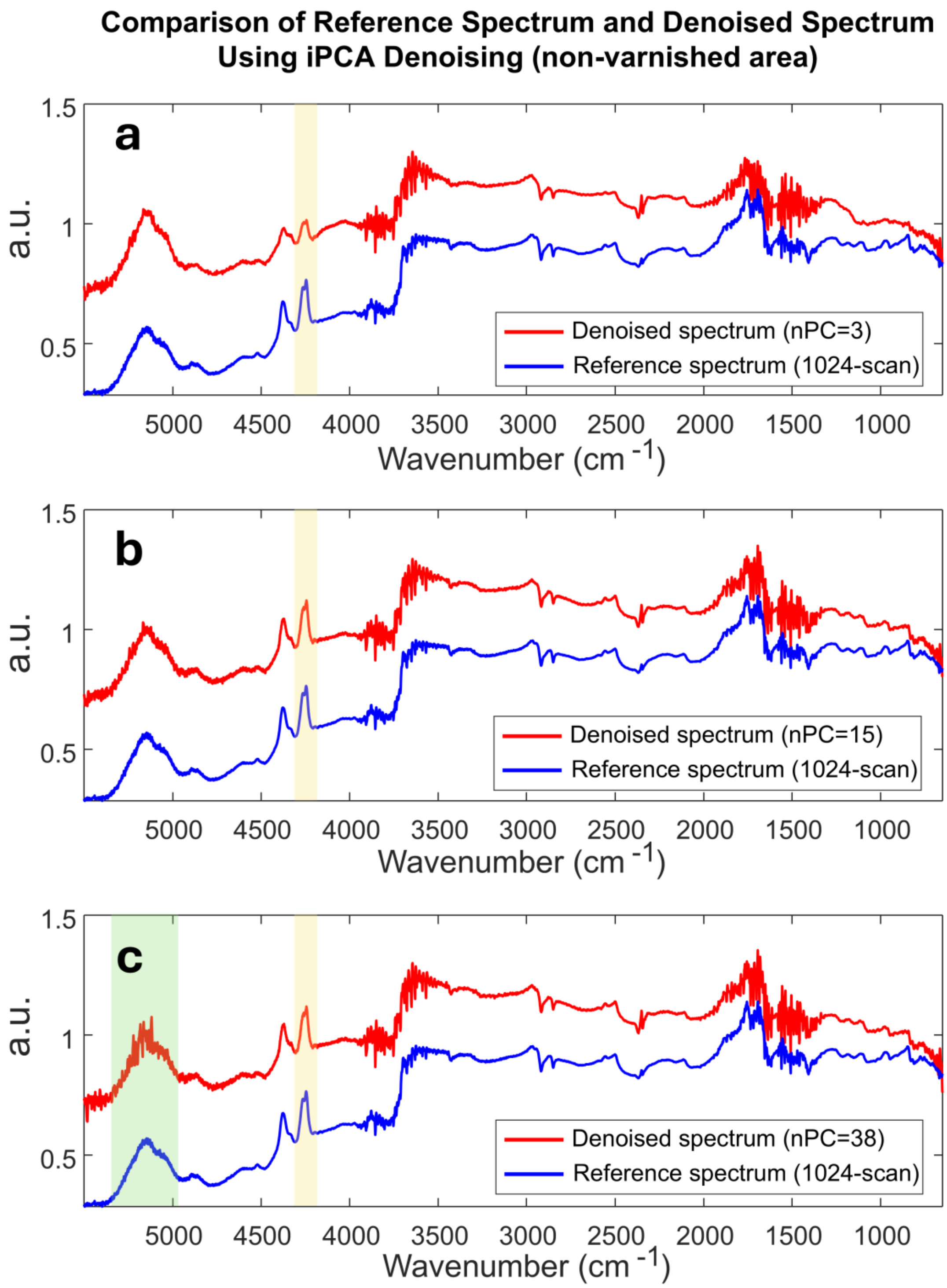

Fig. S4: Comparison of denoised spectra from non-varnished blue area using different number of PCs for inverse PCA denoising.

(a): Comparison of reference spectrum (blue) with denoised spectrum (red) using iPCA (3PCs), resulted in dACOS=0.78 and dRMSE=1.10. The distorted split-peaks of azurite highlighted in yellow.

(b): Comparison of reference spectrum (blue) with denoised spectrum (red) using iPCA (15PCs), resulted in dACOS=0.91 and dRMSE=1.11. The preserved split-peaks of azurite highlighted in yellow.

(c): Comparison of reference spectrum (blue) with denoised spectrum (red) using iPCA (38PCs), resulted in dACOS=0.92 (highest) and dRMSE=1.99. The preserved split-peaks of azurite highlighted in yellow and the increased noise highlighted in green.

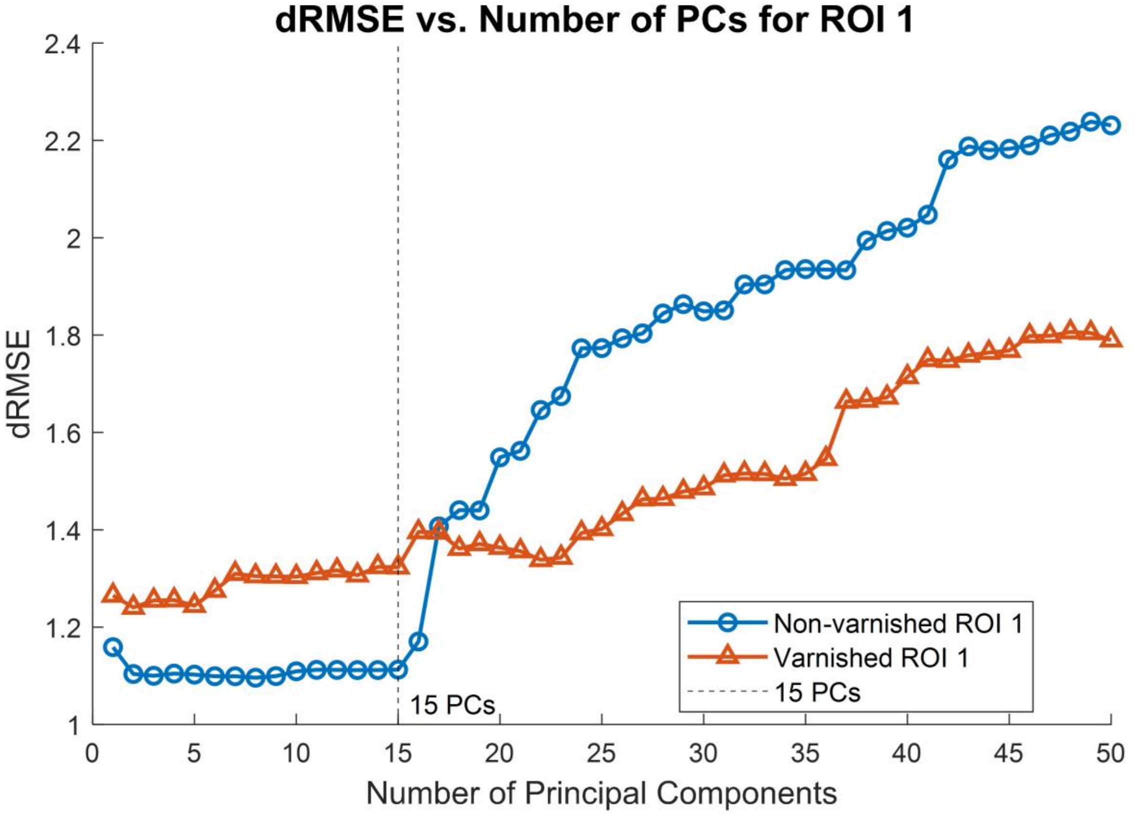

Fig. S5: The trends of dRMSE values across different numbers of PCs used for ROI 1 in non-varnished and varnished areas. The value gradually increased with PC numbers, and showed a distinct jump at approximately 15 PCs.

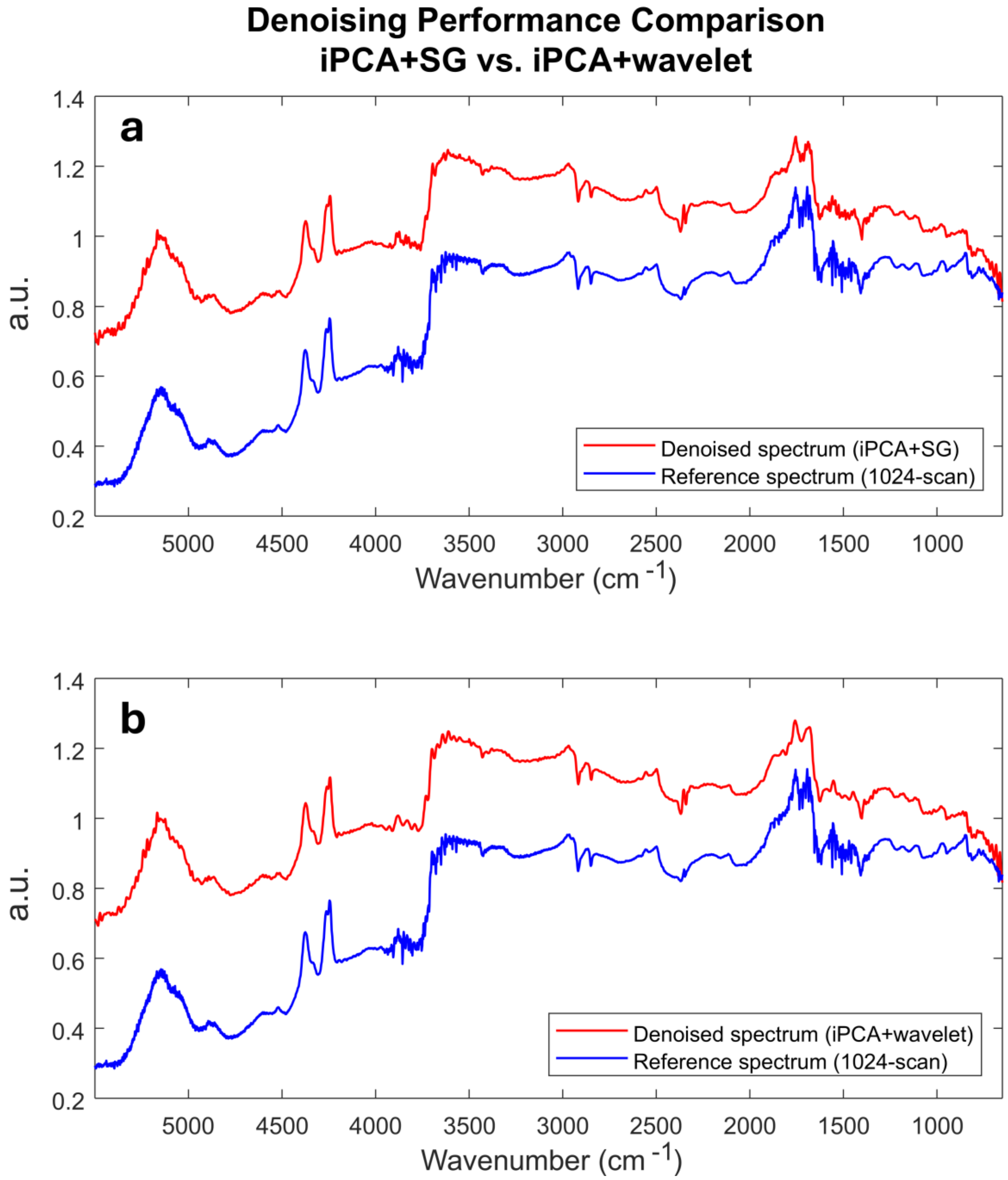

Fig.S6: Comparison of representative single-pixel spectra from the non-varnished area processed with the final two-step denoising methods:

(a) iPCA (15 PCs) + SG smoothing (2nd order polynomial, window 21 for water-vapor regions; window 9 for other regions; 2nd-order polynomial);

(b) iPCA (15 PCs) + symlet order 16 (level 3 for water-vapor regions; level 2 for other regions).

Table S1: Summary of evaluation metrics values for the raw and denoised spectra compared to the 1024-scan reference in ROI 1.

**Method & Parameters**	**Surface Condition**	**ACOS**	**dACOS**	**dRMSE**
Raw	Non-varnished	0.91	0.92	3.67
	Varnished	0.91	0.83	3.11
SG (order=2 window=13)	Non-varnished	0.92	0.85	1.19
	Varnished	0.92	0.77	0.99
SG (order=2 window=29)	Non-varnished	0.92	0.72	0.63
	Varnished	0.92	0.67	0.61
Wavelet (symlet 6 Level 2)	Non-varnished	0.92	0.83	1.74
	Varnished	0.92	0.74	1.32
Wavelet (symlet 6 Level 3)	Non-varnished	0.92	0.76	0.66
	Varnished	0.92	0.72	0.71
Inverse PCA (3 PCs)	Non-varnished	0.91	0.78	1.10
	Varnished	0.92	0.68	1.25
Inverse PCA (15 PCs)	Non-varnished	0.92	0.91	1.11
	Varnished	0.92	0.83	1.32
Inverse PCA (38 PCs)	Non-varnished	0.92	0.92	1.99
	Varnished	0.92	0.85	1.66
Inverse PCA (15 PCs)	Non-varnished	0.92	0.92	0.62
+ SG (order=2 window=9)	Varnished	0.92	0.83	0.76
Inverse PCA (15 PCs)	Non-varnished	0.92	0.92	0.57
+ wavelet (symlet 16 Level 2)	Varnished	0.92	0.83	0.69

Table S2: Experimental wavenumbers and band assignments for the painting materials studied in this work.

Compound	Experimental wavenumber (cm^{-1})	Assignment	
Azurite	~1880	$\nu_1+\nu_4$ (CO_3^{2-})	[1]
	2497	$\nu_1+\nu_3$ (CO_3^{2-})	[1]
	2560	$\nu_1+\nu_3$ (CO_3^{2-})	[1]
	2600	$\nu_1+\nu_3$ (CO_3^{2-})	[1]
	4245	ν (OH) + νs (CO_3^{2-})	[2]
	4262	ν (OH) + νa (CO_3^{2-})	[2]
	4374	$\nu + \delta$ (OH)	[1]
Egg binder	1680 (maximum)	Amide I	[3]
	1560 (maximum)	Amide II	[3]
	1750 (maximum)	ν (C=O)	[3]
	2800-3000	ν (C-H)	[3]
	~3300	ν (N-H)	[3]
	4260	νs(CH) + δ(CH)	[4]
	4333	νa(CH) + δ(CH)	[4]
Dammar	1392	δ CH	[5]
	1483	δ CH	[5]
	1735 (sh)	ν (C=O)	[5]
	1768 (maximum)	ν (C=O)	[5]
Gypsum	2115	ν (SO_4^{2-})	[6]
	2223	$2\nu_3$ (SO_4^{2-})	[6]
	5058 (sh)	$\nu + \delta$(OH)	[4]
	~5150	$\nu + \delta$(OH)	[4]
Sienna	~1100	νa (Si-O)	[1]

*Note: References for assignments

[1]: C. Miliani *et.al.*, 2012, doi: 10.1007/s00339-011-6708-2

[2]: S. Wu *et.al.*, 2024, doi: 10.3390/cryst14090791

[3]: F. Rosi *et al.*, 2009, doi: 10.1007/s00216-009-3108-y

[4]: M Vagnini *et al.*, 2009, doi: 10.1007/s00216-009-3145-6

[5]: C. Invernizzi *et al.*, 2018, doi: 10.1155/2018/7823248

[6]: F. Rosi *et al.*, 2010, doi: 10.1366/000370210792080975

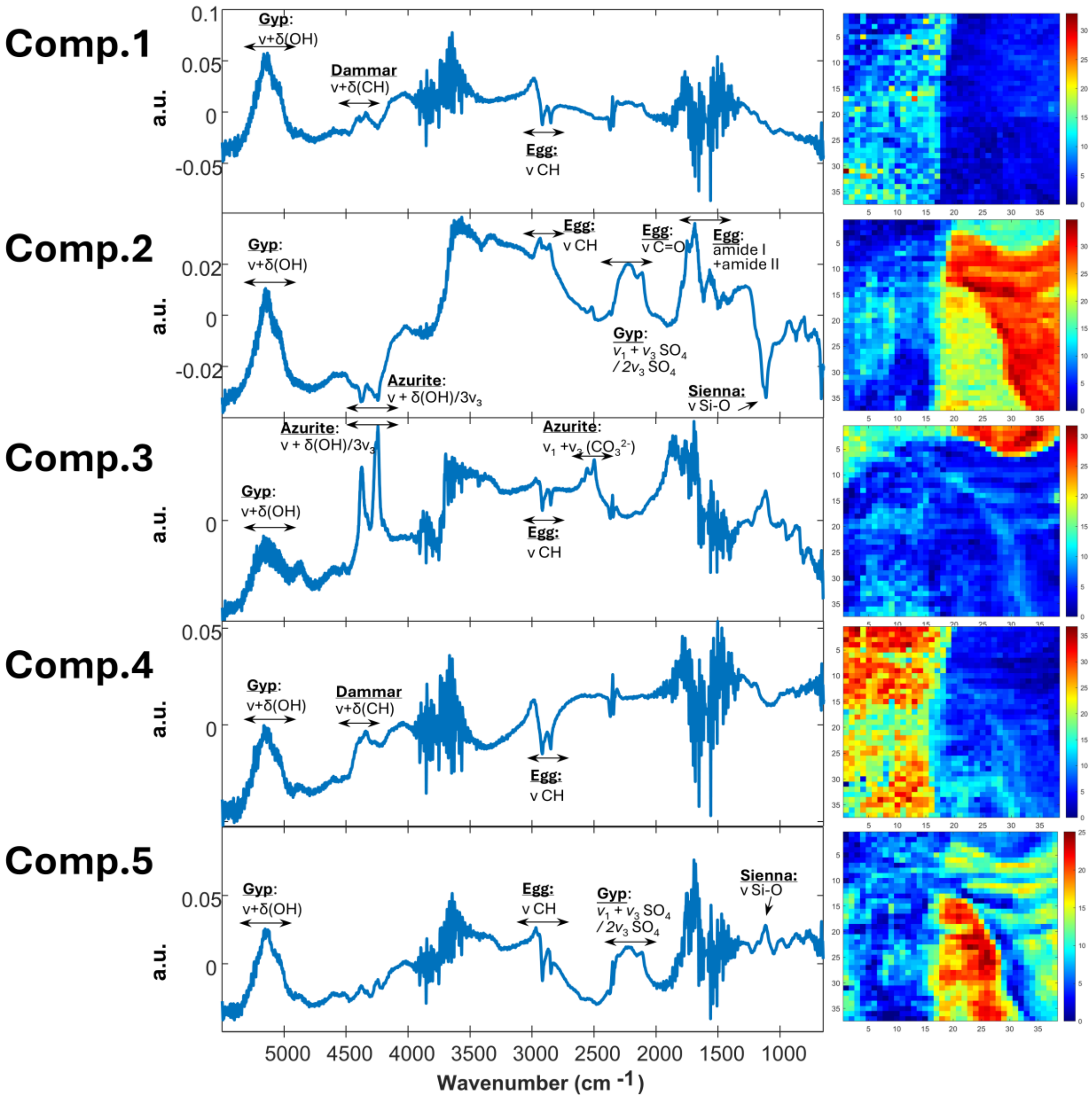

Fig. S7: MCR-ALS results obtained on raw data after SNV correction (no denoising). Five components with their corresponding resolved spectral profiles and concentration maps are presented. (*Gyp=gypsum)
